# Experimental Peritonitis

**Published:** 1887-08

**Authors:** 


Experimental Peritonitis.—Dr. B. Pernice publishes, in *La Revista Internazionali di Med., e. Chirurg.*, an account of his recent experiments instituted to determine the nature and causation of peritonitis. The following is a summary of his conclusions:

1. The injection even of very small quantities of nitric, chlorhydric, sulphuric, phenic and acetic acids, of corrosive sublimate and of nitrate of silver, into the abdominal cavity, are capable of producing peritonitis and even perforation.

2. The peritonitis thus induced is usually of a sero-fibrinous variety, sometimes serous, but never purulent

3. Micrococci are constantly found in the peritoneal exudations. These are principally diplococci and may be easily cultivated in gelatin.

4. These bacteria are possibly derived from the blood, since we have detected them in the blood of healthy animals. They also abound in the air where the animals are kept.

5. Pure cultures of these diplococci are not capable of inducing peritonitis, either when injected in the blood, the peritoneal cavity, or in the cellular tissues.

